# Response Surface Modelling Nafion-117 Sorption of Tetraammineplatinum(II) Chloride in the Electroless Plating of IPMCs

**DOI:** 10.3390/polym16162338

**Published:** 2024-08-18

**Authors:** Eyman Manaf, Golnoosh Abdeali, Sean Reidy, Clement L. Higginbotham, John G. Lyons

**Affiliations:** 1 PRISM Research Institute, Technological University of the Shannon, N37 HD68 Athlone, Ireland; a00278637@student.tus.ie (E.M.); golnoosh.abdeali@tus.ie (G.A.); clem.higginbotham@tus.ie (C.L.H.); 2 Faculty of Science & Health, Technological University of the Shannon, N37 HD68 Athlone, Ireland; sean.reidy@tus.ie; 3 Faculty of Engineering & Informatics, Technological University of the Shannon, N37 HD68 Athlone, Ireland

**Keywords:** ionic polymer–metal composite, electroless plating, Nafion-117, thin films, platinum

## Abstract

This work looks at the effects of a varying concentration, soak time, pH and temperature on the sorption of tetraammineplatinum(II) chloride (Pt-Ammine) in Nafion-117 films in the context of the electroless plating of ionic polymer–metal composites (IPMCs). Sorption is characterised by atomic absorption spectroscopy. A definitive screening design carried out determined all four factors to be significant for further analysis using response surface modelling. A duplicated central composite design (CCD) was utilised to characterise how the four factors affect the sorption amount and efficiency. Regression models for both responses were of poor fit. Nevertheless, key insights were obtained on the effects of the process parameters on sorption behaviour. The results indicate that above 0.5 g/L Pt-Ammine sorption, the platinisation of 10 × 50 mm IPMC samples through sodium borohydride reduction becomes redundant by the surface resistance metric. IPMCs with surface resistance values of approximately 2.5 Ω/square were obtained through only one round of chemical reduction. Varying surface morphologies and electrode thicknesses were analysed under a scanning electron microscope. The CCD parameter settings were validated. Recommended settings for optimised Pt-Ammine sorption in 10 × 50 mm Nafion-117 films were identified as follows: 1.0 g/L Pt-Ammine concentration, 24 h soak time, pH of 3 and temperature of 20 °C.

## 1. Introduction

Ionic polymer–metal composites (IPMCs) are polymer–metal composites known for their ion-exchange capabilities [1,2]. They are a subset of electroactive polymers whereby they deform in the presence of an electric field [1]. They have a wide variety of applications as IPMCs can act as both sensor and/or actuator (e.g., micro-robots, artificial muscles, viscometer) [3]. The general structure of IPMCs consists of a polymer membrane, commonly Nafion (a perfluorinated sulfonic-acid ionomer developed and trademarked by DuPont), sandwiched between electrode layers (noble metals such as platinum are commonly used). There exists a variety of methods in producing IPMCs. Conventionally, the preferred chemical method of manufacturing IPMCs is through electroless plating due to the good adhesion produced between the electrodes and the polymer membrane [4]. No standardised method has been established for the electroless plating of IPMCs due to a lack of fundamental understanding of how its myriad process parameters affect IPMC performance. The effects of process parameters such as the concentrations of reagents used, stirring time, soaking time, temperature and pH on how they impact the quality and performance of IPMCs have not been fully explored, particularly with reference to their interconnectivity. Generally, the electroless plating of IPMCs in the literature follows four main stages: pre-treatment, ion-exchange, reduction and post-treatment. This article will explore and characterise the effects of the concentration, soak time, pH and temperature on the Nafion-117 sorption of tetraammineplatinum(II) chloride, the ion-exchange stage in the electroless plating of IPMCs.

### Electroless Plating of Ionic Polymer–Metal Composites (IPMCs)

**Figure 1:**
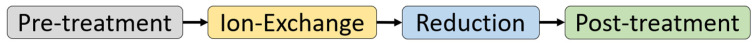
Steps in the electroless plating of IPMCs.

The conventional and preferred chemical method of manufacturing IPMCs is through electroless plating due to good bonding between the electrodes and the Nafion membrane [4]. Electroless plating is a controlled autocatalytic deposition of a continuous layer on a catalytic surface in the presence of a complex compound and reducing agent [5]. The chemical deposition of platinum onto Nafion was first described in a 1980 patent by H. Takenaka and E. Torikai [6]. Known as the Takenaka–Torikai method, the plating utilised tetraammineplatinum(II) chloride (Pt-Ammine) as the metal complex and sodium borohydride as the primary reducing agent. The platinisation was performed by suspending the Nafion membrane between two chambers, one side filled with the metal complex, the other with the reducing agent. This method was surpassed by Fedkiw et al. in 1989 after the introduction of an impregnation–reduction method, which involved soaking the Nafion in the metal complex to exchange ions, and using sodium borohydride to reduce the impregnated Pt atoms [7]. Despite non-standardisation, with reported methods varying in terms of process parameters such as the concentrations of reagents used, stirring time, soaking time and many more, the electroless plating of IPMCs generally follows four main stages: pre-treatment, ion-exchange, reduction and post-treatment (as given in Figure 1).

The ion-exchange process involves exposing the membrane to the metal complex solution, Pt-Ammine [Pt(NH_3_)_4_Cl_2_] or hexaammineplatinum(IV) chloride [Pt(NH_3_)_6_Cl_4_], typically through soaking the membrane in the solution. This is to allow the platinum ions to diffuse into the Nafion membrane [8,9]. The platinum particles are adsorbed onto the Nafion and platinised in the next stage of reduction. Reported soaking times range from 30 min to 24 h, with most of the literature not specifying why those durations were chosen. Similarly, varied concentrations of the metal complex solution can be found in the literature. K. Oguro recommends more than 3 mg/cm^2^ area of the Nafion membrane [10]. Others such as Yang et al. and Kim et al. use 0.5 g/L and 0.02 M, respectively [8,9]. In addition to that, many have stated the inclusion of ammonia solution in the soaking process for pH adjustment, but no pH value was specified. Several works have looked at optimising the different stages of the electroless plating of IPMCs. One prominent work is the study performed by Kim et al. examining the effect of 13 factors on the performance of IPMCs (characterised by the maximum force produced) through a Taguchi design [8]. The 13 factors encompass various factors from all of the stages in the electroless plating of IPMCs (as in Figure 1). However, in the ion-exchange stage studied, only the platinum salt concentration and stirring time and how those factors affected the force produced were considered. The sorption kinetics of platinum salt uptake into the membrane and the effects of the temperature and pH were not assessed. By contrast, a study by Nasef et al. did address the sorption kinetics of metals in Nafion-117 membranes—not of platinum but of heavy metals [11]. The effects of the concentration, contact time, pH and temperature, known drivers of sorption kinetics, were explored with regard to the amount of heavy metals adsorbed per mg of the membrane. However, a one-factor-at-a-time (OFAT) approach was utilised instead of design of experiments (DOE). This means that the interactions of the four factors cannot be accurately and fully estimated with regard to the sorption kinetics. The different configurations of the ion-exchange stage in the electroless plating of IPMCs found in the literature are summarised in Table 1.

## 2. Materials and Methods

### 2.1. Atomic Absorption Spectroscopy (AAS)

AAS is used to quantify Nafion-117 uptake of Pt-Ammine. After soaking the Nafion films in the known concentrations of Pt-Ammine solutions, concentrations of the remaining solutions were measured using a Varian AA240FS atomic absorption spectrometer (Varian, Palo Alto, CA, USA). A Photron platinum hollow cathode lamp (Photron, Tokyo, Japan), Agilent-coded (P840C), was used as the radiation source. Little literature exists on the AAS of the Pt-Ammine compound. Several preliminary test runs were carried out to determine the optimal testing parameters for the spectrometer. The settings specified in Table 2 were used for all testing involving AAS.

Both standard and sample solutions were adjusted to pH 3 for AAS analysis, and the sample solutions were diluted down to be between 10 and 100 mg/L. This is because the limit of detection was found to be below 10 mg/L and going above 100 mg/L was found to exhibit deviations in absorbance signals between the pH-adjusted sample and standard solutions.

#### Gage Repeatability and Reproducibility Analysis

The reliability of the measuring system utilised is important in ensuring good and consistent results. Measurement system analysis is typically used in determining measurement accuracy and in identifying sources of error. In this case, the absorption values generated from the atomic absorption spectrometer can be sensitive to a multitude of factors, and day-to-day measurement variation needs to be determined to ensure accurate results.

A triplicate Gage Repeatability and Reproducibility (Gage R&R) study was carried out measuring the absorbance values of 6 different concentrations of Pt-Ammine solution: 25 mg/L, 50 mg/L, 100 mg/L, 200 mg/L, 400 mg/L and 800 mg/L. Each day over 3 days, the average of 3 absorbance readings for each concentration was recorded. The ‘operator’ factor was left out since the absorption measurement using the AA spectrometer is a fairly automated process with all the parameter inputs being handled by the SpectrAA software, SpectrAA 5.1 PRO. The Gage R&R analysis was carried out in Minitab 20.2.

### 2.2. Design of Experiments (DOE)

DOE is a branch of statistical analysis involved in the systematic design, analysis and optimisation of experiments [21]. In the case of studying how multiple parameters affect a yield response, DOE is advantageous in providing the fewest number of experimental runs for the most amount of data, grounded on statistical principles [21].

Nafion-117 uptake of a platinum complex is not yet fully understood. These 4 factors were analysed as to how they affect the Nafion-117 membrane uptake of Pt-Ammine: concentration of platinum complex, soak time, pH and temperature.

#### Definitive Screening Design (DSD)

A screening design was first carried out to identify the most significant factors. Common screening designs include 2-level fractional factorials, Placket–Burman designs and DSDs [22]. According to the sparsity-of-effects principle, most processes are mainly dominated by main effects and low-order interactions (i.e., 2-way interactions), as opposed to higher-order interactions (i.e., 3-way interactions and beyond) [23]. DSD was chosen due to it being a resolution IV design where main terms are not confounded [24]. The analysed factors and their respective levels are given in Table 3. The upper and lower limits of the factor levels were chosen based on the range of configurations presented in the previous literature (refer Table 1).

The uncoded DSD design matrix is given in Table A1. All the DOE and statistical analysis were carried out using Minitab 20.2. Nafion-117 strips were soaked in the solutions configured according to Table A1. They were then removed and kept in sample bags. The remaining solutions from the DSD were diluted down and Pt-Ammine concentrations were determined from AAS.

### 2.3. Sample Preparation

#### 2.3.1. IPMC Preparation

**Figure 2:**
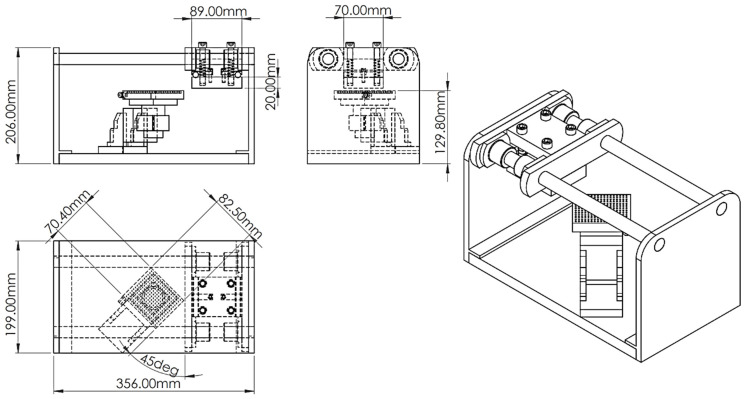
Nafion membrane sanding apparatus schematic.

Nafion-117 films (177.8 µm thickness), consisting of a fluorocarbon backbone, polytetrafluoroethylene (PTFE), copolymerised with a pendant ionic group, polysulfonyl fluoride vinyl ether (SO_3_^−^), were commercially obtained from Ion Power Gmbh (Munich, Germany). They were cut into strips of 10 × 50 mm. The strips were roughened using a 400-grit sandpaper on a specially built hand-sander (Figure 2), 150 cycles on each side of the membrane (forward and backwards counts as one cycle), employing the grid method [15,25]. The membranes were then ultrasonicated using a Clifton MU−14 ultrasonic bath for 30 min with deionised water as the medium. They were then boiled in 2 M hydrochloric acid (HCl) and deionised water for 30 min, respectively.

#### 2.3.2. Solution Preparation

The different solutions were made up to the specified concentrations in the DSD with pH adjustment accounted for. The Pt-Ammine solutions (tetraammineplatinum(II) chloride powder obtained from Sigma-Aldrich, St. Louis, MO, USA) were made from diluting an 8.5 g/L Pt-Ammine solution down to the respective concentrations of 0.5 g/L, 4.25 g/L and 8 g/L.

pH Adjustment:

The pH of the solutions was adjusted using 0.1 M and 0.01 M HCl and sodium hydroxide (NaOH). A Hach SensION 1 with 2-point calibration was used to monitor pH changes. The solutions were only initially adjusted to the desired pH using a 1 mL bulb pipette and were not kept constant. HCl and NaOH were chosen as the acid–base due to them fully dissociating in water: HCl into H^+^ and Cl^−^ ions, and NaOH into Na^+^ and OH^−^ ions. It was decided against pH buffers due to the multitude of compounds present in those, and not knowing specifically the effects of those compounds on the adsorption process. They can be used, but thorough analysis must be performed for different buffers to identify the effects of the multiple compounds present on the sorption process.

Temperature Adjustment:

The temperatures of the solutions were adjusted using BINDER BD 115 incubators. The incubators were set to 30, 40, 50 and 60 °C, respectively, and were allowed to reach the set temperatures. The Pt-Ammine solutions, covered with parafilm, were then placed in the incubators and allowed to equilibrate for 150 min, before placing the Nafion-117 strips in them. For solutions set to the temperature of 20 °C, they were placed on a benchtop at room temperature with the test tubes covered with parafilm.

**Figure 3:**
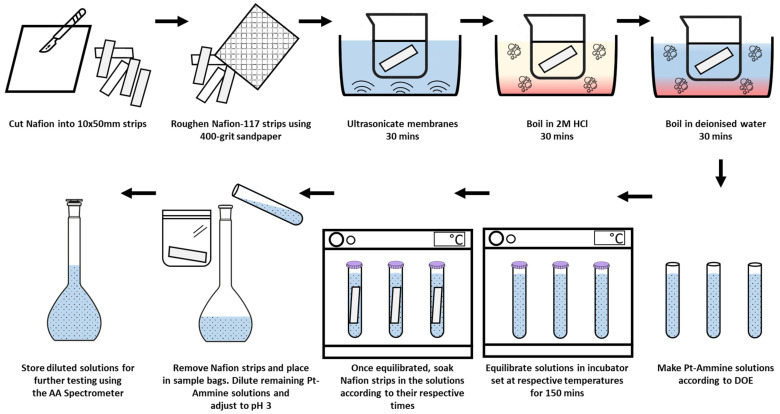
Summarised sample preparation steps for DOE.

Dilution for AAS Testing:

After soaking the Nafion-117 strips in the DSD-configured solutions, the strips were removed, and the solutions were diluted down for further testing using AAS. As shown earlier, matrix interference on the absorbance signal of the Pt-Ammine solutions due to pH adjustments is minimal within the 0 to 100 mg/L linear range. Thus, DSD solutions were diluted down to within the range of up to no less than 10 mg/L to 100 mg/L since detecting concentrations below 10 mg/L proved to be unreliable and inconsistent at the specified settings in Table 2. A summarised methodology of the sample preparation is shown in Figure 3. For solutions made up to the 8 g/L and 4.25 g/L concentrations, a dilution factor of 100 was chosen, whilst for the 0.5 g/L solutions, a dilution factor of 10 was chosen to ensure the concentration in the remaining solution did not fall below the 10 mg/L concentration, which was the limit of detection calculated during spectrophotometry method development.

### 2.4. Surface Resistance Measurement

**Figure 4:**
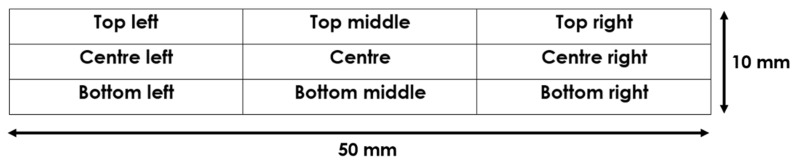
Regions for surface resistance measurements.

Surface resistance was measured using an Ossila Four Point Probe. The IPMC samples were divided into 9 regions, as shown in Figure 4, and an average of 100 surface resistance measurements were taken for each region.

### 2.5. Scanning Electron Microscopy (SEM)

Surface and cross-sectional morphologies were examined through a TESCAN MIRA XMU (Tescan, Brno, Czech Republic) with 20 kv voltage. InBeam detector was used for cross-section morphology analysis whilst back-scattered electron (BSE) detection was used for surface morphology. For cross-section analysis, the samples were hand-cut using microtome blades. They were then sputter-coated with gold using an Agar Sputter Coater, at a current of 30 mA and sputter time of 55 s per run. Cross-sectional samples were double-coated (total sputter time of 110 s) whilst surface samples were coated once. Samples were adhered to sample holders by carbon tape. After exposing prepared samples to electron beam radiation in vacuum condition, images were captured at various magnifications. Energy Dispersive X-ray (EDAX) analysis was employed alongside SEM imaging for elemental mapping of the IPMCs.

## 3. Results and Discussion

### 3.1. Gage R&R Analysis

Results for the Gage R&R analysis are given in Table 4 and Table 5. As mentioned in the methodology section, since the ‘operator’ factor was excluded, only repeatability is accounted for in the analysis. From Table 4, the variation contribution of repeatability accounts for only 0.03% of the total variation, with the remaining variation coming from part-to-part. This is relatively low and within the acceptable range of being less than 10%.

Similarly, looking at Table 5, total variation due to the measurement system, i.e., Total Gage R&R is 1.63%, is acceptable since it is less than 10%. From these results, we can conclude that the absorption values measured using the AA spectrometer are good and consistent, and the measured parts contribute to most of the variation in the study instead of the measurement system, making this measurement system reliable.

### 3.2. Definitive Screening Design (DSD) Results

The design matrix and results from the DSD, for both the sorption amount and sorption efficiency, are given in Table A1.

#### 3.2.1. Sorption Amount-DSD

**Figure 5:**
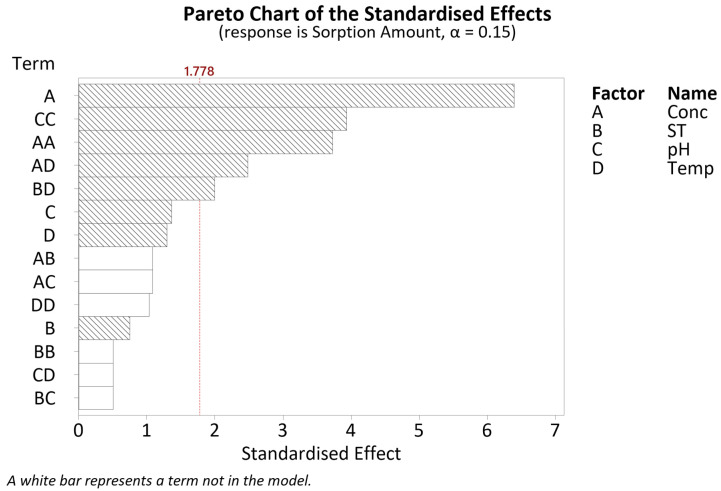
DSD Pareto chart of the standardised effects for sorption amount.

After a series of model reductions through a stepwise approach, a regression model with an R^2^ value of 95.51% was obtained. The ANOVA results are given in Table A2. Examining the Pareto chart given in Figure 5, the significant factors include the linear term for concentration (A), the square terms for concentration (AA) and pH (CC), and the interactions as follows: concentration*temperature (AD) and soak time*temperature (BD). The (*) symbol indicates an interaction between terms.

Despite the other linear terms, soak time (B), pH (C) and temperature (D), not being statistically significant, they are included as to adhere to a hierarchical model. This implies that all the four factors screened are significant to be further examined through surface response modelling.

#### 3.2.2. Sorption Efficiency-DSD

**Figure 6:**
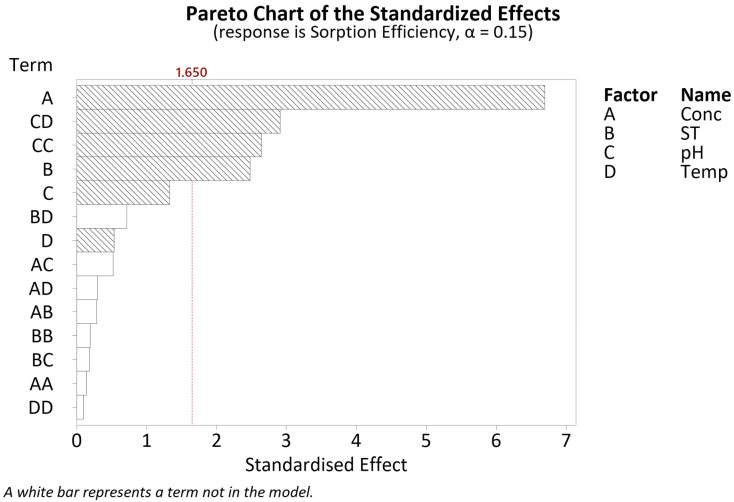
DSD Pareto chart of the standardised effects for sorption efficiency.

Similarly, with regard to the sorption efficiency, after a series of model reductions through a stepwise selection, a regression model with an R^2^ value of 91.95% was obtained. The ANOVA results are given in Table A3. Looking at the Pareto chart in Figure 6, statistically significant factors include the linear terms for concentration (A) and soak time (B), the interaction term for pH*temperature (CD) as well as the square term for pH (CC).

Adhering to model hierarchy, this indicates that all the four terms screened with regard to the sorption efficiency are relevant for further evaluation using response surface modelling.

## 4. Parameter Optimisation and Characterisation

### 4.1. Response Surface Methodology (RSM)

After subsequent screening of the factors through DSD, all the four factors, concentration, soak time, pH and temperature, were deemed to be important factors for further analysis using RSM. A central composite design (CCD) was employed to further study the four factors. The CCD with 31 runs was duplicated, and the design matrix and results are given in Table A4. The low and high levels from Table 3 were inserted into the CCD as axial points. Blocking was applied on replication. The duplicated CCD resulted in 62 samples.

**Figure 7:**
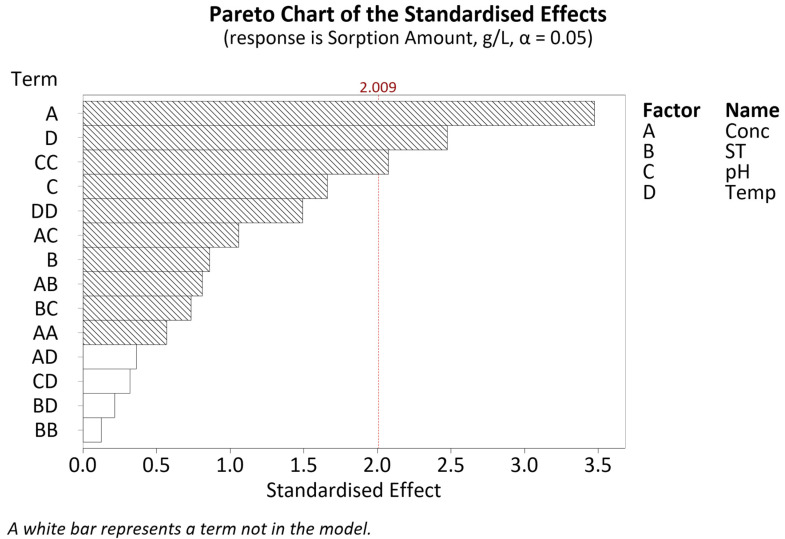
CCD Pareto chart of the standardised effects for sorption amount.

The CCD results were input into Minitab 20.2 to generate response surface regression models for two responses: sorption amount and sorption efficiency. The regression equations for both responses and their respective R^2^ values after model reduction are given in Table 6. The terms included in both models can be inferred from the Pareto charts given (Figure 7 and Figure 10).

#### 4.1.1. Sorption Amount-CCD

The regression model for the sorption amount only produced an R^2^ value of 49.09%. This implies a not-so-well-fitted model, supported by a significant lack-of-fit *p*-value of 0.008, as shown in Table A5. This signifies that only approximately 49% of the variation in the sorption amount can be explained by the varying concentration, soak time, pH and temperature. This testifies the complex nature of Nafion sorption kinetics and isotherms. As discussed by Kusoglu et al. [2], ion transport in Nafion is governed by the properties of water and how they interact with the sulfonate acid sites in the membrane, as well as the properties of the polymer chains in structuring mesoscale transport networks. Despite the poor fit, the results from the regression model could give further insights into the complex interactions.

**Figure 8:**
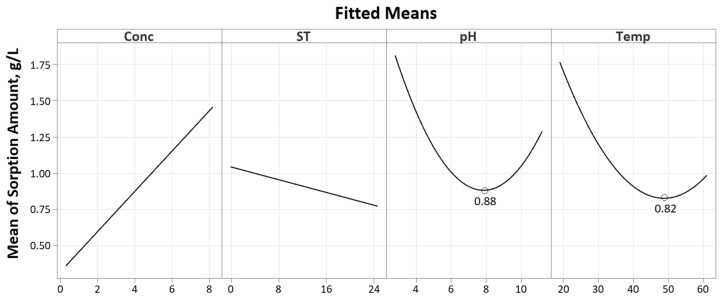
Main effects plot for sorption amount.

Referring to the Pareto chart given in Figure 7, only three factors are significant: concentration, temperature and the square term for pH (CC). The pH square term signifies a quadratic response, as evident in the main effects plot given in Figure 8.

Concentration

Concentration is a significant factor affecting the sorption amount of the Pt-Ammine with a *p*-value of 0.001 (as shown in Table A5). A linear relationship is observed between the initial concentration of the Pt-Ammine and the adsorbed amount. The SO_3_H sites have a higher affinity for higher valency ions as well as a preference for metal cations as compared to protons [2,11,26]. As the Pt-Ammine solution has a higher initial concentration of the [Pt(NH_3_)_4_]^2+^ ion compared to the membranes, increasing the initial concentration from 0.5 g/L to 8 g/L increases the concentration gradient, resulting in increased sorption.

Soak time

Referring to the Pareto chart in Figure 7 and Table A5, the soak time bears no significance with regard to the Pt-Ammine sorption amount (*p*-value of 0.394). Similarly, any of its interactions are also of no significance. Going from a soak time of 30 min to 24 h, the mean sorption amount trends downwards, i.e., sorption decreases with the soak time. The expected response was that sorption would increase with the soak time and level off after some time as it reaches maximum sorption such as reported by Nasef et al. in the Nafion adsorption of heavy metals [11]. However, in that particular case, the investigated concentrations only lie within the range of several mg/L as compared to the much higher concentrations investigated in this study. A possible explanation for this behaviour would be maximum adsorption consecutively followed by desorption.

pH

**Figure 9:**
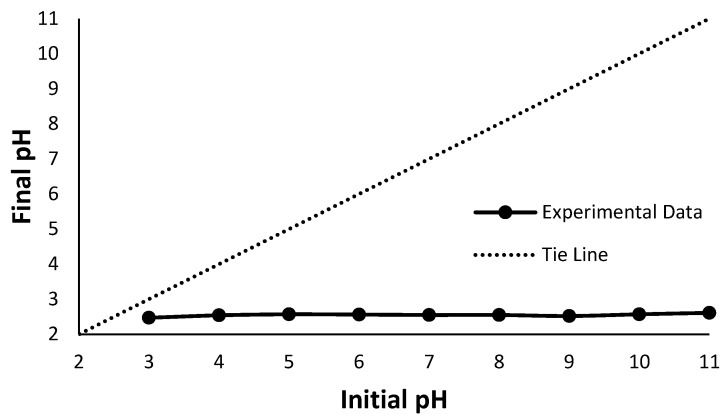
PZC determination through pH drift at 0.5 g/L.

Solution pH plays an important role in the sorption since it controls the surface charge density of both the sorbent and analyte, affecting their electrostatic interactions [27]. The square term for pH (CC) is significant with a *p*-value of 0.043. This implies a curved mean sorption yield, as evident from the main effects plot in Figure 8. At a pH of 3, the mean sorption is at a maximum. This could be attributed to SO_3_H sites in Nafion having higher affinity for higher valency ions as well as a preference for metal cations as compared to protons [2,11,26]. As the pH increases, the mean sorption amount decreases until slightly above the pH of 7 after which the response starts trending upwards until pH 11. In the previous literature, researchers added varying concentrations of ammonia solution in the impregnation process for pH adjustment purposes [12,13]. Experimentally, the Pt-Ammine solution was found to be approximately around a pH of 5. The addition of the ammonia solution would push this value closer to 7 or even slightly beyond that into alkaline territory. Rashid et al. adjusted the solution pH to 12 using 0.1 M NaOH. At a more basic pH where the OH^−^ ion concentration is higher, there is less competition between the Pt-Ammine and hydronium ions (H_3_O^+^) at adsorption sites, leading to more sorption of Pt-Ammine [11]. A point-zero-charge (PZC) experiment was carried out to further explain the curved response in pH. A pH drift method was utilised whereby solutions of Pt-Ammine were made to be between the pH of 3 and 11 at a 0.5 g/L concentration. Samples of Nafion-117 (10 × 50 mm) that were ultrasonicated, preboiled in 2M HCl and deionised water were soaked in 15 mL, 0.5 g/L Pt-Ammine solutions for 24 h. The Nafion-117 strips were then removed, and the final pH was measured. The results for the PZC are graphed in Figure 9. A tie line was drawn (dashed line) where initial pH equals final pH, and the intersection between the tie line and experimental data line is the PZC.

Evident from Figure 9, the Pt-Ammine solutions at varying pH levels dropped to a constant pH value of approximately 2.5, indicating the H^+^ ions in the protonated Nafion-117 films have been displaced into the solution. However, this does not help explain the quadratic pH response indicated by the regression model. A possibility might be the inapt testing methodology for PZC, or a different metric might be better suited to explain the response such as isoelectric point [28].

Temperature

Referring to the main effects plot in Figure 8, it is evident that the temperature has a significant effect on the sorption amount (supported by a *p*-value of 0.017 as given in Table A5). Going from a temperature of 20 °C to approximately 50 °C, an increase in the temperature results in a steep decrease in the mean sorption amount. However, as the temperature is further increased up to 60 °C, the sorption trends slightly upwards. Ion and water transport in Nafion membranes are highly interrelated since ion transport is dependent on solvent [2]. The literature is divided on the exact effects of the temperature on the sorption properties of Nafion. As concluded by Sánchez [29], the temperature effects on the Nafion sorption capacity are still not clear—whether it be an increasing or decreasing function of the temperature. This lack of clarity is partly due to the effects of varying thermal histories of Nafion membranes, such as pre-drying, preboiling and annealing, on the sorption kinetics and thermodynamics [2,30,31,32]. A study by Kusoglu et al. inferred that the liquid water uptake of preboiled Nafion membranes is relatively independent of the temperature [30]. Similarly, Nasef et al. found that the sorption of heavy metals in preboiled Nafion-117 membranes does not change with the temperature [11]. The steep decline in the sorption amount with the temperature indicates a physisorption mechanism between the Pt-Ammine ions and the sulfonate acid sites in the Nafion membrane since physisorption is an exothermic process [33]. This implies that weak electrostatic forces (Van der Waals) are involved in the sorption process. As the temperature increases, more energy is supplied, allowing for easier breakage of these intermolecular forces. This is also supported by the fact that sorbed species in Nafion membranes can be desorbed through washing with solvents, as demonstrated by Nasef et al. whereby Nafion membranes were regenerated by 0.1 M HNO_3_ treatment for 16 h at 25 °C [11]. Chemisorbed species cannot readily desorb under ambient temperature conditions [34]. However, another aspect that requires consideration is that an increase in the temperature would also increase the rate of ion diffusion into the membrane. This might explain the increase in mean sorption after approximately 50 °C.

#### 4.1.2. Sorption Efficiency-CCD

**Figure 10:**
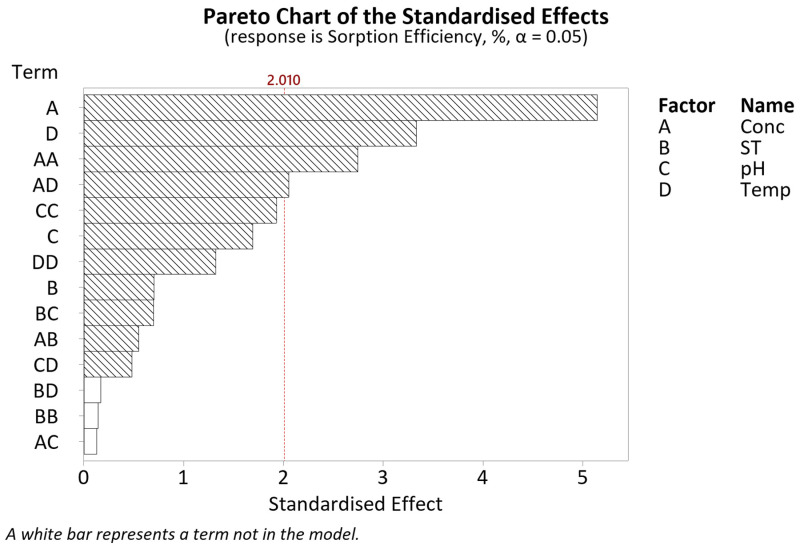
CCD Pareto chart of the standardised effects for sorption efficiency.

The sorption efficiency was obtained by dividing the measured sorption amount by the initial concentration of Pt-Ammine solution. Referring to Table 6, the regression model for sorption efficiency has a higher R^2^ value as opposed to the sorption amount, implying better model fitting as compared to the latter response. However, the significant lack-of-fit *p*-value of 0.012 from Table A6 indicates this model to be a poor fit. Approximately 58% of the variation in sorption efficiency can be described by the terms included in the model. Analysing the Pareto chart for sorption efficiency given in Figure 10, it is evident that four terms are significant: the linear terms of concentration (A) and temperature (D), the square term of concentration (AA) and the interaction between the concentration and temperature (AD).

Concentration

**Figure 11:**
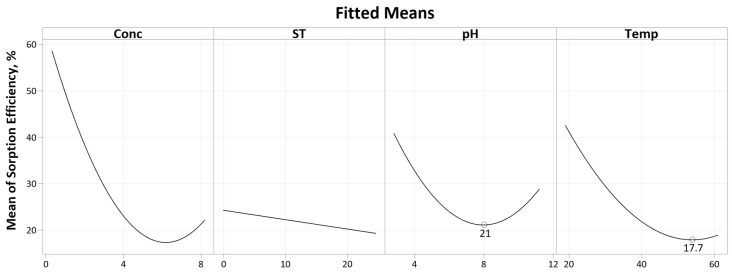
Main effects plot for sorption efficiency.

Referring to the Pareto chart in Figure 10, both the linear and square terms of concentration are significant. Based on the main effects plot in Figure 11, the sorption efficiency decreases, going from a concentration of 0 to 6 g/L. After that point, the efficiency starts increasing again. It should be noted that the regression model points to a significant interaction between the concentration and temperature (AD), meaning they are dependent on each other. A possible explanation for the decreased sorption efficiency with the increasing concentration is the reduced availability of SO_3_^−^ sorption sites. At high concentrations, the Pt-Ammine aggregates reduce the contact surface area between the adsorbent and adsorbate, resulting in less sorption and a drop in the sorption efficiency. At higher concentrations, the concentration gradient would overcome this, resulting in more sorption and an increase in the sorption efficiency.

Soak time

Similar to the sorption amount, the soak time is also not a significant factor in modelling the mean sorption efficiency. From Figure 11, the soak time exhibits a slight decline in the mean sorption efficiency, going from a soak time of 30 min to 24 h. This can be attributed to the decrease in the sorption amount with the increasing soak time, attributed to a possible adsorption process followed by a desorption process.

pH

The model suggests that the pH linear term is not a significant factor (*p*-value of 0.097) in describing the sorption efficiency of Pt-Ammine in Nafion-117. Similarly, all interactions involving pH are also of no significance, as can be seen in Table A6.

It is evident from the R^2^ values and the lack-of-fit significance that the models do not adequately describe the data. Despite exhaustive attempts of factor and response transformations, the model showed no significant improvements. This implies that a second-degree polynomial CCD model cannot fit the response [24]. It is important to note that a CCD is only an approximation at best. A higher-order model is needed to better fit the data set, possibly a cubic or quartic model. Absence of higher-order interactions in the regression model could be the explanatory variable that would help better explain the variance in response with regard to the four studied factors producing better R^2^ values.

Temperature

Temperature is a significant factor in the sorption efficiency yield, with the mean sorption efficiency decreasing as the temperature increases from 20 °C to 60 °C. This is in line with the exothermic physisorption process—less sorbed amount with increasing temperature results in less efficient sorption, i.e., decreasing sorption efficiency.

### 4.2. Surface Resistance Measurement Results

A common metric used to quantify the quality of electrodes produced on IPMCs is through surface resistance measurements [15,35,36,37]. Several studies have shown a negative correlation between the blocking force and surface resistance, i.e., a lower surface resistance results in a higher blocking force [8,13,17,38]. To see if the sorption amount produces a significant difference in platinisation from the reduction of IPMCs using NaBH4, six samples were chosen, three from each replicated run, classified into a low, medium and high sorption amount, as specified in Table 7.

The samples were reduced in 100 mL of 5% NaBH_4_ for 1 h at 60 °C. The samples were then blotted with tissue paper and kept in silica gel overnight to remove moisture. The measured surface resistance values, given in Ω/square, are given in Table 8.

Comparing the surface resistance values for the three levels of sorption specified, the low sorption runs (Runs 17 and 48) showed significant differences as compared to the other two levels. The surface measurement values for Runs 17 and 48 are also very inconsistent across sample regions, ranging from values of approximately 12 Ω/square up to 2000 Ω/square.

The differences between the medium sorption levels (Runs 18 and 49) and high sorption levels (Runs 3 and 34) are minor. Furthermore, it can be observed that the surface resistance values are approximately consistent throughout all the sample regions for Runs 3, 18, 34 and 49.

**Figure 12:**
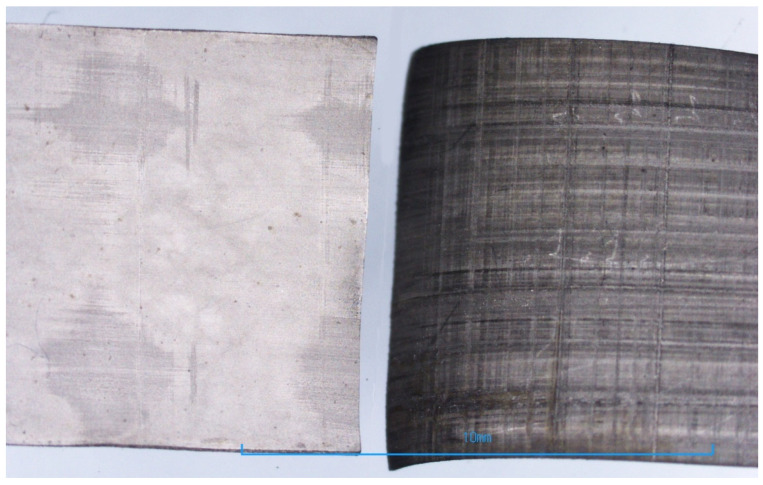
Visual difference between Run 34 (**left**) and Run 48 (**right**).

A visual difference is observed between samples with low and high surface resistance values. Figure 12 compares appearances between Run 34 and Run 48. A distinct colour difference is evident, with Run 34 exhibiting a silver electrode colour whereas Run 48 shows a darker shade of grey. Run 34 has a consistent surface measure reading averaging approximately 2.5, as evident from Table 8, whereas Run 48 has a surface resistance measurement ranging from 11 Ω/square to 9000 Ω/square.

This seems to indicate that above a sorption value of approximately 0.5 g/L for the 10 × 50 mm film, the platinisation of the Nafion-117 film through NaBH_4_ reduction becomes redundant by the metric of surface resistance. This has been discussed by Rashid et al. in which they found that after a certain point, the surface resistance reached a limiting value of 1.8 Ω/square [20]. The limiting factor here could be the availability of the borohydride ions in the reduction process, as given in Equation (1) [9].

(1) $${\mathrm{NaBH}}_{4}+4{\left[\mathrm{Pt}{\left({\mathrm{NH}}_{3}\right)}_{4}\right]}^{2+}+8{\mathrm{OH}}^{-}\to 4{\mathrm{Pt}}^{0}+16{\mathrm{NH}}_{3}+{\mathrm{NaBO}}_{2}+6H_{2}O$$

Stoichiometrically, nine moles of borohydride ions are needed to reduce four moles of the Pt-Ammine [39]. Limited availability of borohydride ions, which would prevent effective platinum reduction, could be due to NaBH_4_ hydrolysing to produce hydrogen gas [40]. To test this hypothesis, two runs with approximately consistent high sorption (above 0.5 g/L) measured across the two replicates, Run 5 and Run 36, were chosen to be further reduced in 100 mL of 10% NaBH_4_ for 1 h at 60 °C. The measured surface resistances by region are given in Table 9.

Despite doubling the NaBH_4_ concentration, the surface resistance values of Runs 5 and 36 indicate no significant differences as compared to the previous samples, potentially ruling out borohydride being the limiting factor in the platinisation.

It is worth noting the low surface resistance values obtained in Runs 3, 18, 34 and 49 are similar to the values measured by Yip et al. and Kim et al. [8,13]. However, their electroless plating processes involved several rounds of coating through the use of several reducing agents: sodium borohydride, hydrazine monohydrate and hydroxylamine hydrochloride. This study demonstrates the same surface resistance values can be obtained from only one round of coating through sodium borohydride reduction (5% concentration, 1 h stir time at 60 °C) by having the Nafion-117 films (10 × 50 mm in size) adsorb approximately 0.5 g/L of Pt-Ammine.

**Figure 13:**
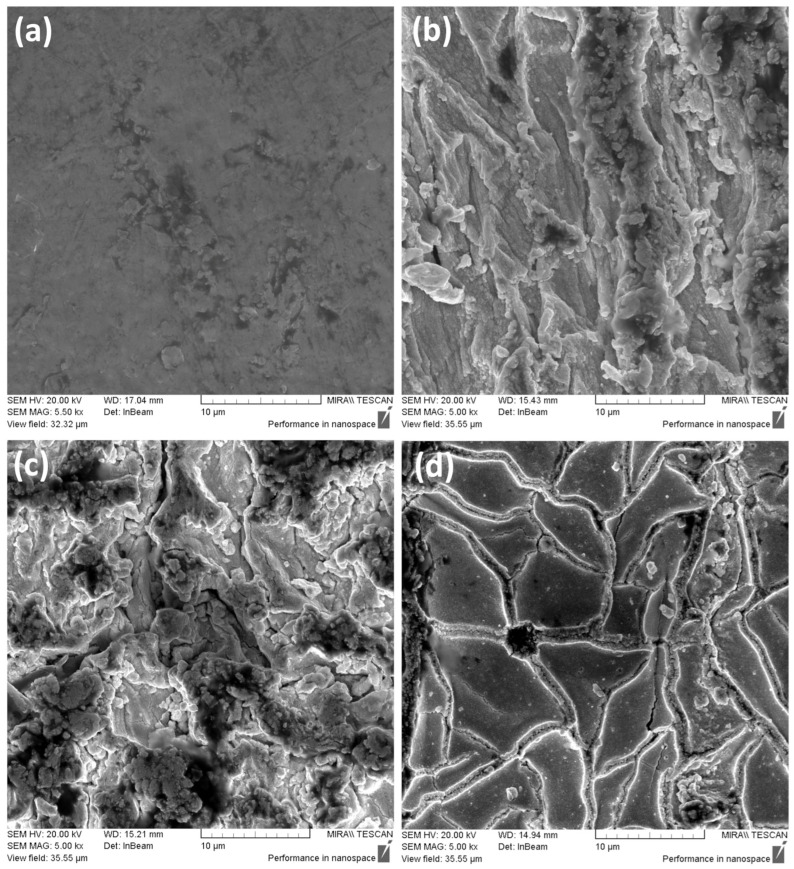
Surface SEMs of IPMCs at 5 k magnification, descendingly sorted by surface resistance—(**a**) Run 48, (**b**) Run 17, (**c**) Run 5 and (**d**) Run 3.

As evident from the SEMs given in Figure 13, discernible surface morphologies can be seen at different surface resistance values. Comparing the different surfaces, the surface SEM of Run 48 in Figure 13a shows a somewhat smooth surface, with no noticeable structures, suggesting no presence of platinum growth. This absence is most likely what is causing high surface resistance values in some regions of Run 48. Moving to Run 17 in Figure 13b, platinum growth is visible, but not as aggregated as the surface of Run 5 seen in Figure 13c. Interestingly, the surface morphology for Run 3 in Figure 13d exhibits a dense and homogenous platinum layer distribution, with crack formations. Several studies in the literature attributed high surface resistance to crack formations, which leaves gaps in the electrode layer, disrupting the interconnectedness of the platinum particles [13,20,37]. 

**Figure 14:**
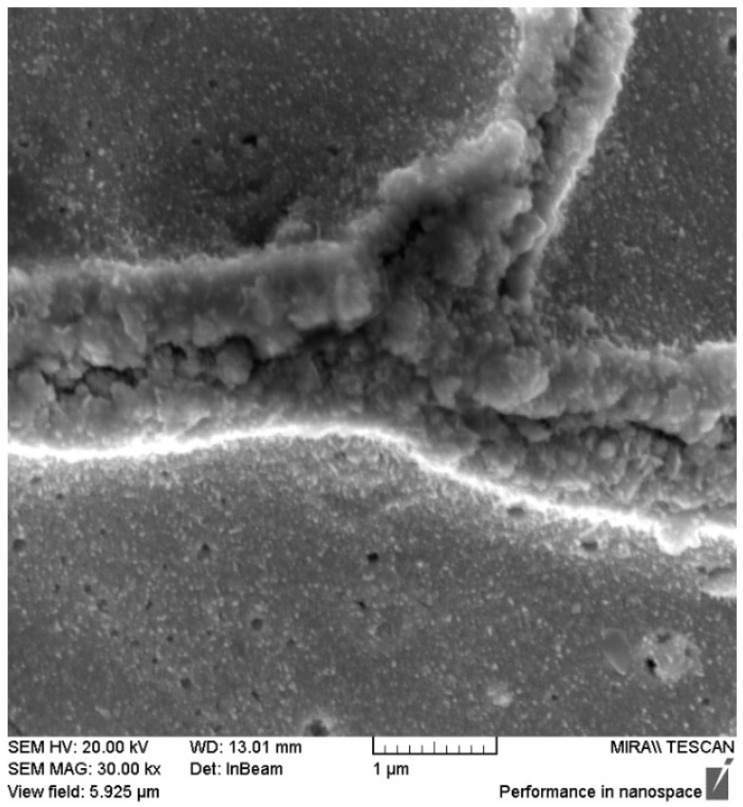
SEM of Run 3 crack formation at 30 k magnification.

However, despite the crack formations in Run 3, further magnification of the cracks, seen in Figure 14, reveals that they are still connected, giving rise to low surface resistance values.

EDAX analysis was carried out on the surface of the samples. The weight percentages of fluorine and platinum for the different runs are given in Table 10. Comparing the weight percentages of fluorine, Run 48 has the highest value at approximately 23.42%. This signifies a larger percentage of exposed Nafion membrane area where there is little to no deposition of platinum. This is further supported by the platinum weight percentage of Run 48 being the lowest at approximately 27.73%. This is in line with the morphology seen in Figure 13a.

**Figure 15:**
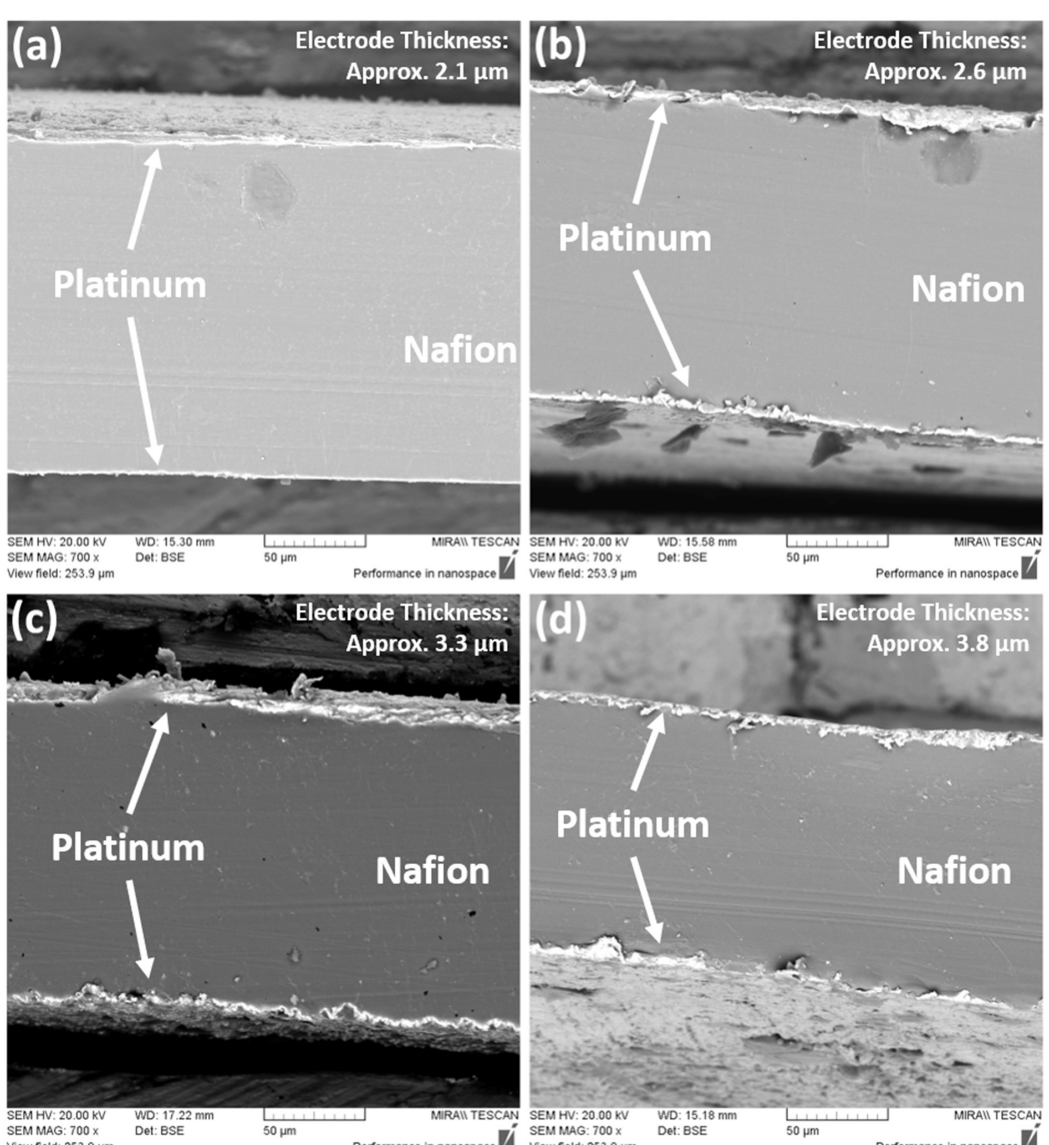
Cross-sectional SEMs of (**a**) Run 48, (**b**) Run 17, (**c**) Run 5 and (**d**) Run 3 at 700 magnification.

The platinum weight percentage for Run 5 is relatively high as compared to Runs 3 and 17. This could be an overestimation in the EDAX analysis since surface topography (e.g., ridges, valleys) can affect signal attenuation [41,42]. The cross-sections of the samples were also investigated under SEM, as shown in Figure 15. Clear distinction between the polymer and metal layer can be seen (as indicated in Figure 15), with the large darker region being the Nafion layer, and the lighter layer being the platinum layer.

The thicknesses of the platinum electrodes for Runs 48, 17, 5 and 3 were determined to be approximately 2.1 µm, 2.6 µm, 3.3 µm and 3.8 µm, respectively. It could be inferred that a thicker electrode layer equates to denser platinum deposition, resulting in lower surface resistance values, as metal clusters on the ionomer surface transform from isolated agglomerates to a uniform, fully covered film [3,15,20,43,44].

### 4.3. Response Surface Modelling Validation

Referring to the main effects plots for both the sorption amount and sorption efficiency, despite the not-so-well-fitted response surface model, the response seems to indicate a high sorption amount and high sorption efficiency at higher concentrations of Pt-Ammine, lower pH and lower temperature with the soak time not indicating significant effects on both of the responses. To verify this, three samples were made according to the following settings: concentration of 1.0 g/L, soak time of 24 h, pH of 3 and temperature of approximately 20 °C. The sorption results are given in Table 11.

All the samples recorded more than 0.5 g/L sorption, with the lowest sorption efficiency being 64.28% and the highest being 93.37%. The huge discrepancy in the sorption amount and efficiency between the samples could be attributed to either dilution inaccuracies or non-uniform membrane properties between samples. As demonstrated earlier, above a 0.5 g/L sorption, the platinisation of the IPMCs becomes redundant by the surface resistance metric and excellent values were obtained. Thus, it could be concluded that for the platinisation of the 10 × 50 mm IPMC samples, the recommended configurations for the optimised Nafion-117 sorption of Pt-Ammine are as follows: 1.0 g/L Pt-Ammine concentration, 24 h soak time, pH of 3 and temperature of approximately 20 °C.

### 4.4. Limitations and Recommendations

This study mainly looked at the effects of external parameters (concentration, pH, soak time, temperature) on the membrane sorption amount and efficiency of Pt-Ammine. However, membrane properties such as their ion-exchange capacity and degree of saturation could also influence the responses. This necessitates further investigation considering the membrane properties.

A potential challenge faced was the evaporation of the Pt-Ammine solution, more specifically the evaporation of water, leaving behind a more concentrated Pt-Ammine solution. Especially for the mass production of IPMCs where big batches of the Pt-Ammine solution are involved, an evaporation study is vital to ensure no significant inaccuracies with the concentrations of the Pt-Ammine.

Another issue with the process is the possible contamination of the Pt-Ammine solution with more Pt-Ammine when using the same pipettor for the whole process. It is recommended to use single-use pipette tips to avoid cross-contamination. If using a single pipette tip for the whole process, ensure to rinse the pipette with deionised water between each sample. Since the process deals with high concentrations of Pt-Ammine, even small amounts of contamination could cause the final reading to be inaccurate, especially in the dilution process. This would lead to AA readings detecting higher concentrations of Pt-Ammine than the initial concentration.

The pre-treatment process involving the surface roughening of the Nafion membranes could further be improved. The built apparatus only provides a uniform platform to roughen the Nafion surface using sandpaper without considering the pressure applied. An ideal roughening apparatus would have a tuneable pressure mechanism to allow for uniform pressure distribution throughout the sanding process. This is vital since the amount of material removed from the thin membranes impacts adsorption sites, varying the sorption and plating amount.

The RSM applied is aimed at optimising the electroless plating of IPMCs. However, as evident from the R^2^ values of the regression models, the four factors studied do not fully describe the variation in the sorption yield. The authors conclude from these findings that a more exploratory design of experiments be used to further explore the relations between the four factors in modelling the sorption response yield, such as the use of a full factorial design. However, this will cost more runs, with a three-level four-factor full factorial resulting in 81 runs for a single replicate design.

## 5. Conclusions

The effects of the concentration, soak time, pH and temperature on the Nafion-117 sorption of tetraammineplatinum(II) chloride in the electroless plating of IPMCs were explored through response surface modelling (RSM). Despite the poor-fit models for both responses, sorption amount and sorption efficiency, the RSM provided key insights into the sorption behaviour with regard to the four factors studied. With regard to the mean sorption amount, an increase in the Pt-Ammine concentration results in a sorption amount increase. The soak time going from 30 min to 24 h shows no significant changes. Both the pH and temperature responses exhibited quadratic responses with regard to the sorption amount. Possible explanations for their behaviour were discussed. Looking at the sorption efficiency, an increase in the concentration shows a drop in the sorption efficiency, whereas not much change can be seen with an increase in the soak time. Both the pH and temperature responses for the sorption efficiency show a similar quadratic behaviour to their sorption amount counterparts. Possible reasonings for this were discussed. This study found that above a 0.5 g/L sorption, the platinisation of the 10 × 50 mm IPMCs by sodium borohydride reduction becomes redundant by the surface resistance metric and excellent values were obtained, comparable to measurements reported in the literature. However, the excellent surface resistance values reported in this study were obtained through only one round of coating, as compared to the literature having to utilise multiple rounds of coating and multiple reducing agents to yield similar results. Varying surface resistance values were attributed to discernible surface morphologies and differing electrode layer thicknesses. The recommended configurations for the optimised Nafion-117 sorption of Pt-Ammine in the platinisation of IPMCs are as follows: 1.0 g/L Pt-Ammine concentration, 24 h soak time, pH of 3 and temperature of approximately 20 °C.

## Figures and Tables

**Table 1:** Ion-exchange configurations found in the literature.

Platinum Complex Solution Impregnation					
Author(s)	Membrane Size	Concentration	Soak Time	pH Adjustment	Temp. °C
Kim et al., 2003 [8]	5 × 5 cm	NS ^1^	>1 h	5% NH_4_OH ^2^	NS
Shahinpoor, 2015 [12]	5 × 5 cm	NS	2–4 h	30% NH_4_OH	40–60
Yip et al., 2011 [13]	50 × 60 mm	0.5 g/L	Overnight	5% NH_4_OH	NS
Liu et al., 1992 [14]	1 in sides hexagon	0.5–1.6 mM (0.17–0.54 g/L)	24 h	NS	50
Tian et al., 2021 [15]	30 × 10 mm	0.01 M (3.34 g/L)	12 h	NS	NS
Palmre et al., 2014 [16]	50 × 10 mm	15–25 mM (5.01–8.35 g/L)	3–4 h	NS	NS
Kim et al., 2022 [17]	0.5 × 2.5 cm	0.02 M (6.68 g/L)	3.5 h	NS	NS
Yang et al., 2020 [9]	10 × 50 mm	2 g/L	24 h	NS	NS
Oguro et al., 2000 [10]	30 cm^2^	2 g/L	>3 h (usually overnight)	5% NH_4_OH	NS
Xu et al., 2021 [18]	30 × 29 mm	3 mg/mm^2^	14 h	NS	NS
Ma et al., 2020 [19]	30 × 30 mm	0.5 wt%	24 h	NS	NS
Rashid et al., 2013 [20]	15 mm dia. circle	5–40 mM	3 h	pH 12 (0.1M NaOH)	5, 25, 50

^1^ NS denotes not specified, ^2^ NH_4_OH is ammonium hydroxide.

**Table 2:** AAS configurations.

Variable	Settings
Wavelength	265.9 nm
Instrument Mode	Absorbance
Measurement Mode	Integrate
Slit Width	0.2 nm
Lamp Current	5 mA
Background Correction	BC Off
Measurement Time	5.0 s
Pre-Read Delay	5.0 s
Flame Type	Air/Acetylene
Air Flow	13.50 L/min
Acetylene Flow	2.0 L/min
Burner Height	13.5 mm

**Table 3:** Process variables and their levels for the screening design.

Screening Design Factor Configurations				
Factors	Units	Notation	Factor Levels	
			Low	High
Concentration of Platinum Complex	g/L	Conc.	0.5	8
Soak Time	hours	ST	0.5	24
pH	-	pH	3	11
Temperature	°C	Temp.	20	60

**Table 4:** Variance components for Gage R&R.

Source	Variance Components (VarComp)	% Contribution (of VarComp)
Total Gage R&R	0.0000020	0.03
Repeatability	0.0000020	0.03
Part-To-Part	0.0073146	99.97
Total Variation	0.0073165	100.00

**Table 5:** Gage evaluation.

Source	Standard Deviation (SD)	Study Var. (6 × SD)	% Study Variation
Total Gage R&R	0.0013981	0.008388	1.63
Repeatability	0.0013981	0.008388	1.63
Part-To-Part	0.0855254	0.513152	99.99
Total Variation	0.0855368	0.513221	100.00

**Table 6:** Regression equations and R^2^ values for sorption amount and efficiency.

Response	Uncoded Regression Equation	R^2^ (%)
Sorption Amount	3.22 + 0.498 Conc + 0.0580 ST − 0.378 pH − 0.1003 Temp − 0.0110 Conc*Conc + 0.0355 pH*pH + 0.001021 Temp*Temp − 0.00677 Conc*ST − 0.0260 Conc*pH − 0.00575 ST*pH	49.09
Sorption Efficiency	175.4 − 22.58 Conc + 1.09 ST − 8.31 pH − 2.80 Temp + 1.199 Conc*Conc + 0.741 pH*pH + 0.0203 Temp*Temp − 0.103 Conc*ST + 0.226 Conc*Temp − 0.122 ST*pH − 0.050 pH*Temp	58.05

**Table 7:** Chosen runs for further reduction using NaBH_4_.

Run	Conc. (g/L)	Soak Time (Hour)	pH	Temp. (°C)	Sorption Amount (g/L)	Sorption Level
17	0.5	12.25	7	40	0.285	Low
48	0.5	12.25	7	40	0.278	Low
18	8	12.25	7	40	0.538	Medium
49	8	12.25	7	40	0.498	Medium
3	2.375	18.125	5	30	1.340	High
34	2.375	18.125	5	30	1.295	High

**Table 8:** Surface resistance values for Runs 3, 17, 18, 34, 48 and 49.

**Low Sorption (Ω/Square)**					
Run 17			Run 48		
15.474	39.171	60.931	11.011	8395.465	19995.370
14.049	29.499	40.764	15.327	639.066	6645.429
12.148	27.452	53.150	16.120	2768.155	9217.344
**Medium Sorption** (**Ω/Square**)					
Run 18			Run 49		
3.071	2.698	3.312	2.366	2.441	2.613
3.138	2.893	3.116	2.400	1.854	3.187
2.876	2.503	2.381	2.440	2.394	2.842
**High Sorption** (**Ω/Square**)					
Run 3			Run 34		
2.559	2.592	2.395	2.676	2.702	2.426
2.360	2.420	2.306	2.891	2.076	2.157
2.227	2.418	2.347	2.693	2.868	2.594

**Table 9:** Surface resistance values for Runs 5 and 36.

Run 5 (Ω/square)			Run 36 (Ω/square)		
4.702	4.254	3.487	3.093	3.238	3.331
5.706	3.538	3.403	2.775	2.793	2.932
4.289	4.097	3.577	2.880	2.853	3.113

**Table 10:** Surface EDAX analysis of Runs 3, 5, 17 and 48.

Element Weight (%)	Run 3	Run 5	Run 17	Run 48
Fluorine	0.77	3.31	6.05	23.42
Platinum	47.62	74.24	50.98	27.73

**Table 11:** Sorption amount and efficiency of validation samples.

Sample	Sorption Amount (g/L)	Sorption Efficiency (%)
1	0.643	64.281
2	0.934	93.371
3	0.917	91.722

## Data Availability

The original contributions presented in the study are included in the article; further inquiries can be directed to the corresponding author.

## Appendix A

**Table A1:** Uncoded DSD matrix and results.

Run	Conc. (g/L)	Soak Time (Hour)	pH	Temp. (°C)	Sorption Amount (g/L)	Sorption Efficiency (%)
1	4.25	24	11	60	1.086	25.559
2	4.25	0.5	3	20	1.135	26.708
3	8	12.25	11	20	0.567	7.085
4	0.5	12.25	3	60	0.428	85.559
5	8	24	7	60	0.874	10.922
6	0.5	0.5	7	20	0.118	23.610
7	8	0.5	11	40	0.804	10.050
8	0.5	24	3	40	0.322	64.351
9	8	0.5	3	60	0.909	11.358
10	0.5	24	11	20	0.391	78.164
11	8	24	3	20	0.734	9.178
12	0.5	0.5	11	60	0.150	30.028
13	4.25	12.25	7	40	0.528	12.427

**Table A2:** Sorption amount—DSD model ANOVA.

Source	DF	Adj SS	Adj MS	F-Value	*p*-Value
Model	8	1.27955	0.159944	10.64	0.018
Linear	4	0.67607	0.169018	11.25	0.019
Conc	1	0.61441	0.614412	40.88	0.003
ST	1	0.00846	0.008463	0.56	0.495
pH	1	0.02804	0.028037	1.87	0.244
Temp	1	0.02516	0.025160	1.67	0.265
Square	2	0.46784	0.233918	15.56	0.013
Conc*Conc	1	0.20873	0.208734	13.89	0.020
pH*pH	1	0.23273	0.232733	15.49	0.017
2-Way Interactions	2	0.16638	0.083190	5.54	0.070
Conc*Temp	1	0.09254	0.092543	6.16	0.068
ST*Temp	1	0.06001	0.060006	3.99	0.116
Error	4	0.06012	0.015029		
Total	12	1.33967			

**Table A3:** Sorption efficiency—DSD model ANOVA.

Source	DF	Adj SS	Adj MS	F-Value	*p*-Value
Model	6	8304.90	1384.15	11.40	0.005
Linear	4	6430.37	1607.59	13.25	0.004
Conc	1	5434.54	5434.54	44.78	0.001
ST	1	746.85	746.85	6.15	0.048
pH	1	214.07	214.07	1.76	0.232
Temp	1	34.90	34.90	0.29	0.611
Square	1	846.38	846.38	6.97	0.039
pH*pH	1	846.38	846.38	6.97	0.039
2-Way Interactions	1	1028.16	1028.16	8.47	0.027
pH*Temp	1	1028.16	1028.16	8.47	0.027
Error	6	728.22	121.37		
Total	12	9033.12			

**Table A4:** Uncoded CCD matrix and results.

Run	Conc. (g/L)	Soak Time (Hour)	pH	Temp. (°C)	Sorption Amount (g/L)	Sorption Eff. (%)
1	2.375	6.375	5	30	1.426	60.061
2	6.125	6.375	5	30	3.229	52.712
3	2.375	18.125	5	30	1.340	56.430
4	6.125	18.125	5	30	3.016	49.234
5	2.375	6.375	9	30	1.234	51.945
6	6.125	6.375	9	30	2.442	39.876
7	2.375	18.125	9	30	1.142	48.101
8	6.125	18.125	9	30	1.895	30.932
9	2.375	6.375	5	50	0.579	24.394
10	6.125	6.375	5	50	2.600	42.443
11	2.375	18.125	5	50	0.787	33.150
12	6.125	18.125	5	50	2.407	39.296
13	2.375	6.375	9	50	0.326	13.715
14	6.125	6.375	9	50	2.153	35.155
15	2.375	18.125	9	50	0.077	3.249
16	6.125	18.125	9	50	0.809	13.209
17	0.5	12.25	7	40	0.285	57.087
18	8	12.25	7	40	0.538	6.730
19	4.25	0.5	7	40	0.735	17.289
20	4.25	24	7	40	0.709	16.692
21	4.25	12.25	3	40	1.550	36.471
22	4.25	12.25	11	40	1.740	40.947
23	4.25	12.25	7	20	1.496	35.208
24	4.25	12.25	7	60	1.277	30.043
25	4.25	12.25	7	40	1.248	29.354
26	4.25	12.25	7	40	1.399	32.912
27	4.25	12.25	7	40	1.184	27.862
28	4.25	12.25	7	40	1.121	26.370
29	4.25	12.25	7	40	1.155	27.174
30	4.25	12.25	7	40	1.453	34.178
31	4.25	12.25	7	40	1.396	32.851
32	2.375	6.375	5	30	1.264	53.208
33	6.125	6.375	5	30	1.312	21.421
34	2.375	18.125	5	30	1.295	54.541
35	6.125	18.125	5	30	1.257	20.517
36	2.375	6.375	9	30	1.169	49.212
37	6.125	6.375	9	30	1.193	19.483
38	2.375	18.125	9	30	1.280	53.875
39	6.125	18.125	9	30	1.059	17.288
40	2.375	6.375	5	50	0.995	41.885
41	6.125	6.375	5	50	1.059	17.288
42	2.375	18.125	5	50	0.995	41.885
43	6.125	18.125	5	50	1.146	18.709
44	2.375	6.375	9	50	0.924	38.888
45	6.125	6.375	9	50	0.909	14.835
46	2.375	18.125	9	50	0.892	37.556
47	6.125	18.125	9	50	0.814	13.285
48	0.5	12.25	7	40	0.278	55.627
49	8	12.25	7	40	0.498	6.222
50	4.25	0.5	7	40	0.560	13.180
51	4.25	24	7	40	0.339	7.969
52	4.25	12.25	3	40	0.592	13.925
53	4.25	12.25	11	40	0.608	14.297
54	4.25	12.25	7	20	0.639	15.042
55	4.25	12.25	7	60	0.442	10.389
56	4.25	12.25	7	40	0.434	10.203
57	4.25	12.25	7	40	0.331	7.783
58	4.25	12.25	7	40	0.275	6.481
59	4.25	12.25	7	40	0.117	2.758
60	4.25	12.25	7	40	1.024	24.090
61	4.25	12.25	7	40	0.971	22.844
62	4.25	12.25	7	40	0.994	23.378

**Table A5:** Sorption amount—CCD model ANOVA (after model reduction).

Source	DF	Adj SS	Adj MS	F-Value	*p*-Value
Model	11	13.0368	1.18516	4.38	0.000
Blocks	1	4.7092	4.70924	17.41	0.000
Linear	4	5.8665	1.46662	5.42	0.001
Conc	1	3.2661	3.26606	12.08	0.001
ST	1	0.1999	0.19994	0.74	0.394
pH	1	0.7445	0.74450	2.75	0.103
Temp	1	1.6560	1.65597	6.12	0.017
Square	3	1.8340	0.61133	2.26	0.093
Conc*Conc	1	0.0872	0.08721	0.32	0.573
pH*pH	1	1.1636	1.16363	4.30	0.043
Temp*Temp	1	0.6030	0.60305	2.23	0.142
2-Way Interactions	3	0.6270	0.20901	0.77	0.515
Conc*ST	1	0.1780	0.17798	0.66	0.421
Conc*pH	1	0.3031	0.30306	1.12	0.295
ST*pH	1	0.1460	0.14601	0.54	0.466
Error	50	13.5218	0.27044		
Lack-of-Fit	38	12.5029	0.32902	3.88	0.008
Pure Error	12	1.0189	0.08491		
Total	61	26.5586			

**Table A6:** Sorption efficiency—CCD model ANOVA (after model reduction).

Source	DF	Adj SS	Adj MS	F-Value	*p*-Value
Model	12	9234.0	769.50	5.65	0.000
Blocks	1	1422.8	1422.80	10.45	0.002
Linear	4	5575.4	1393.85	10.24	0.000
Conc	1	3604.2	3604.24	26.47	0.000
ST	1	67.4	67.40	0.50	0.485
pH	1	390.3	390.30	2.87	0.097
Temp	1	1513.4	1513.45	11.12	0.002
Square	3	1524.3	508.09	3.73	0.017
Conc*Conc	1	1027.4	1027.38	7.55	0.008
pH*pH	1	507.7	507.74	3.73	0.059
Temp*Temp	1	237.9	237.86	1.75	0.192
2-Way Interaction	4	711.5	177.87	1.31	0.281
Conc*ST	1	41.0	41.00	0.30	0.586
Conc*Temp	1	573.0	572.98	4.21	0.046
ST*pH	1	66.0	66.02	0.48	0.490
pH*Temp	1	31.5	31.49	0.23	0.633
Error	49	6671.6	136.16		
Lack-of-Fit	37	6107.6	165.07	3.51	0.012
Pure Error	12	564.1	47.01		
Total	61	15,905.6			
